# Oxidative Stress in Metabolic Disorders: Pathogenesis, Prevention, and Therapeutics

**DOI:** 10.1155/2016/9137629

**Published:** 2016-02-01

**Authors:** Umesh C. S. Yadav, Vibha Rani, Gagan Deep, Rakesh K. Singh, Komaraiah Palle

**Affiliations:** ^1^School of Life Sciences, Central University of Gujarat, Sector 30, Gandhinagar 382030, India; ^2^Department of Biotechnology, JayPee Institute of Information Technology, A-10, Sector 62, Noida 201 307, India; ^3^Skaggs School of Pharmacy and Pharmaceutical Sciences, University of Colorado Denver, 12850 E. Montview Boulevard, Aurora, CO 80045, USA; ^4^Translational Science Laboratory, College of Medicine, Florida State University, 1115 West Call St., Tallahassee, FL 32306-4300, USA; ^5^Department of Oncologic Sciences, USA Mitchell Cancer Institute, 1660 Spring Hill Avenue, Mobile, AL 36604, USA

Metabolic disorder is characterized by dyslipidemia, insulin refractoriness, defective insulin secretion, glucose intolerance, and chronic inflammation which contribute to dysfunctional cellular physiology and cellular redox imbalance. The increased oxidative environment contributes to a chronic inflammatory condition in the body and creates primary risk factor for the development of several diseases such as diabetes, arthritis, cancer, and cardiovascular complications. The increased oxidative stress in obesity and metabolic disorder can in turn cause progression and sustenance of inflammatory condition by upregulating redox signaling pathways and altered gene expression of inflammatory markers creating a vicious cycle.

Many evidences implicate oxidative stress in the metabolic perturbations in obesity, diabetes, and cardiovascular pathogenesis [1, 2]. The oxidative damage and inflammation originate from both our environmental milieu such as chemicals, toxicants, and nutrients and body's own metabolism as metabolic byproducts and intermediate. The understanding of their roles and effects on cellular physiology has led to the development of novel biomarkers and molecular targets which could be useful in devising innovative approaches in the prevention, diagnosis, and treatment of inflammatory and metabolic diseases. Additionally, the continued intensive research in this area is yielding novel information which is vital towards further advancing our understanding in this complex field.

Till recently, the major focus and efforts have been towards employing antioxidant as preventive/therapeutic agent against metabolic disorders, considering the central role of oxidative stress therein. However, several studies including clinical ones have clearly shown that this strategy has not yielded expected outcomes [3, 4]. Alternatively, several studies have suggested that it would be better to target the pathways involved in reactive oxygen species (ROS) generation rather than ROS neutralization by antioxidants [1, 2]. Several studies have also shown that cells own machinery gets overinvolved in the production of ROS and resultant molecular signaling leads to activation of inflammatory signaling and incessant inflammation. Mitochondrial dysfunction led ROS generation, weakening of cellular antioxidant machinery, aberrant activation of NADPH oxidase system, and lipid peroxidation mechanisms are few examples of oxidative stress sources. Several new molecular targets that are affected by oxidative stress and in turn distort the cellular physiology resulting in pathogenesis are reported in this special issue. These targets include NOTCH-1, Pin-1, galectin-3, paraoxonase-1, HMGB-1, and mitochondrial respiratory complexes.

In the obese diabetic patients, excessive uric acid has been shown to induce cardiovascular disease (CVD) through the generation of ROS and subsequent endothelial dysfunction [5]. The epidemiological studies suggest that uric acid is an independent risk factor for developing CVDs, especially in people with diabetes, hypertension, and heart failure [6]. However, the clear molecular targets of uric acids have not yet been identified. In this issue, H. Xie et al. presented that uric acid induces ROS generation and increased the expression of several inflammatory molecules such as IL-6, TNF-alpha, and MCP-1 through Notch-1 pathway. Silencing of Notch-1 reversed these changes, and so did (−)-epigallocatechin-3-gallate (EGCG) treatment via inhibiting Notch-1. Authors concluded that downregulation of Notch-1 by EGCG could be an effective approach to decrease inflammation and oxidative stress induced by uric acid.

In their study, K. Gawlik et al. explored the antioxidant defense biomarkers present in the blood of type-2 diabetes patients. They reported that ferric reducing antioxidant power (FRAP) and uric acid levels were significantly elevated in obese diabetic patients; however, no significant difference was observed in the biomarkers of antioxidant defense system between patients with or without chronic diabetes. They concluded that in the presence of excessive ROS production the antioxidant system tends to crumble as reflected by decreased level of erythrocytes glutathione.

Y. Wang et al. discussed the role of peptidyl-prolyl isomerase (Pin-1), a key protein involved in cell division and many other cellular functions both in yeast and humans, in protection against high-dose alcohol-induced apoptosis in mouse cardiomyocytes. They reported that, on the one hand, high-dose alcohol-induced Pin-1 promotes mitochondrial dysfunction leading to ROS increase, while, on the other hand, Pin-1 suppresses endothelial nitric oxide synthase (eNOS) expression leading to apoptosis of cardiomyocytes. The authors concluded that due to these critical activities in alcohol-stimulated cardiomyocytes Pin-1 could be a potential therapeutic target in alcohol-induced cardiomyopathy.

In a review article, I. Petyaev elaborated the important role of lycopene, a member of tetraterpene carotenoids, in scavenging the lipid radicals, ROS, and nitric oxide affording protective effects against prooxidant species. However, with ageing and increased accumulation of oxidative species in the body, lycopene effectiveness might be depleted leading to type-2 diabetes and increased risk for CVDs. The limited bioavailability of lycopene due to decreased intestinal absorption with aging and its enzymatic and oxidative degradation could be addressed by designing novel nutraceuticals.

Galectin-3 (Gal-3), a member of lectin family and important regulator of cellular metabolism, has been implicated in obesity, type-2 diabetes, heart failure, and cancer [7–11]. S. Menini et al. in their review article discussed the controversial role of Gal-3 in this field. On the one hand, Gal-3 has been found elevated in obese and diabetic patients, while, on the other hand, Gal-3 knockout mice showed increased adiposity and systemic inflammation related with altered glucose homeostasis suggesting negative modulation of nutrition-induced immune responses. On the contrary, a few studies showed decrease in fat mass and body weight in high fat diet-fed Gal-3 knockout mice. Although Gal-3 has emerged as an important prognostic marker for heart failure, fibrotic diseases, and inflammatory pathways, the authors concluded that more investigations are warranted to clarify its role in high fat diet-induced obesity and diabetes.

While presenting association between oxidative stress marker plasma 8-isoprostane and the activity of paraoxonase-1 with coronary artery disease (CAD), A. Kuchta et al. suggested that 8-isoprostane could be an important biomarker of lipid peroxidation. They reported a correlation of decreased activity of paraoxonase-1 with decreased protection against lipid oxidation which could be linked with CAD.

High Mobility Group Box-1 (HMGB-1) is a nuclear protein that regulates the transcription of many genes and also involved in the immune response and inflammation. H. Wu et al. investigated how HMGB-1 protein is associated with diabetes-induced oxidative stress that critically regulates endothelial progenitor cell (EPC) dysfunction. In their study, they showed that advanced glycation end products enhanced HMGB-1 levels in EPCs which was downregulated by antioxidant N-acetylcysteine (NAC), suggesting that HMGB-1 could be an important regulator in diabetes-induced oxidative stress and resultant EPC dysfunction.

A. Keller et al. discussed the critical role of mitochondrial perturbation in vascular smooth muscle cells caused by nutritional stress during diabetes. They explored the premise that nutritional stress in diabetes may impair adaptive mitochondrial plasticity via NOS-mediated pathway. They reported that in Goto-Kakizaki diabetes mouse model high glucose enhanced nitric oxide, ROS, and respiratory control ratio while at the same time decreasing phospho-eNOS, uncoupled respiration and mitochondrial respiratory complex expression. With these findings, the authors suggested that eNOS and mitochondria could be potential drug targets in nutritional stress-induced vascular pathogenesis.

Overall, several new findings have been presented in this special issue which have further advanced our knowledge in this exciting area. Also, these studies suggest that oxidative stress is a critical component of metabolic disorders and sustained research efforts are necessary to unravel the complexity associated with oxidative stress in metabolic disorders and related pathologies.
